# Diversity, population genetics, and phylogeography of selected wild mushrooms

**DOI:** 10.1080/21501203.2015.1076980

**Published:** 2015-08-27

**Authors:** Jianping Xu, Lei Cai

Mushrooms are among the most conspicuous sexual reproductive structures in all life forms. In higher plants and animals, their somatic forms are the dominant structures in their life cycles while their sexual reproductive structures are generally inconspicuous and exist only as part of the somatic bodies. However, in mushroom-forming fungi, their somatic hyphae are typically not observable by the naked eye while the observable parts, the mushrooms that we typically see in nature, are for sexual production and sexual-spore dispersion only.

Over the past several centuries, tens of thousands of mushroom species have been described. These mushrooms can vary significantly in size, shape, and colour, as well as in other physical and chemical properties. Some of these variations have been captured in arts and other recorded human histories since ancient times. In the wild, mushrooms play significant roles in nutrient cycling and in the health of plants and animals (including humans). For wild edible mushrooms, they also contribute significantly to the nutritional needs and economic welfare of many communities in both developing and developed countries. For conspicuous poisonous mushrooms, they can generate fears that often extend well beyond the poisonous ones to include all wild mushrooms. For example, one of the most common warnings you read in forests is “Do Not Eat Wild Mushrooms!”. However, despite the important roles mushrooms play in the environment and in our lives, we know relatively little about their natural history.

In this special issue of *Mycology*, the four reviews provide a snapshot of what we know about the diversity, population genetics, and phylogeography of several representative groups of wild mushrooms. Though the reviewed groups of fungi did not include all recent advances on this topic, the selected mushrooms represent a diversity of species and lifestyles, including ascomycetes (Du et al. 2015; Tang et al. 2015) and basidiomycetes (Tang et al. 2015; Wang et al. 2015; Zhang et al. 2015); edible and poisonous species (Zhang et al. 2015); saprophytes and ectomycorrhizae; and plant-associated and animal-associated fungi (Tang et al. 2015). The discussed geographic scales vary from very fine-scale to large geographic regions. In addition, the molecular markers and analytical approaches captured here reflect the diversity of those in the broad scientific literature.

Together, these reviews suggest several emerging trends in the diversity, population genetics, and phylogeography of mushroom fungi. First, molecular markers, especially those based on DNA sequences from multiple loci, are revealing a huge cryptic genetic diversity within most “known” species of mushroom fungi. Many of these “known” species seem to contain multiple divergent lineages that are largely reproductively isolated from each other in nature. Second, there is robust evidence for sexual recombination in all the examined species, consistent with the important roles that sexual spores play in natural populations of these fungi. Third, geographic separation is an important factor in structuring populations and lineages in mushroom fungi. However, the relative effects of geographic separation on population structure and phylogeography can differ significantly among species. These reviews also suggested future trends of research on these mushroom fungi. With slight modifications, most of these perspectives could equally apply to research on other groups of fungi.

We thank the authors for their contributions to this special issue. We hope that these reviews will help stimulate further research on the diversity, population genetics, and phylogeography of mushroom fungi.
